# Exploring the midgut transcriptome of *Phlebotomus papatasi: *comparative analysis of expression profiles of sugar-fed, blood-fed and *Leishmania major*-infected sandflies

**DOI:** 10.1186/1471-2164-8-300

**Published:** 2007-08-30

**Authors:** Marcelo Ramalho-Ortigão, Ryan C Jochim, Jennifer M Anderson, Phillip G Lawyer, Van-My Pham, Shaden Kamhawi, Jesus G Valenzuela

**Affiliations:** 1 Vector Molecular Biology Unit, Laboratory of Malaria and Vector Research, National Institute of Allergy and Infectious Diseases, National Institutes of Health, Bethesda, Maryland, USA; 2 Center for Tropical Disease Research and Training, University of Notre Dame, Notre Dame, Indiana, USA; 3 Preventive Medicine and Biometrics, Emerging Infectious Diseases, Uniformed Services University of the Health Sciences, Bethesda, Maryland, USA; 4 Intracellular Parasite Biology Section, Laboratory of Parasitic Diseases, National Institute of Allergy and Infectious Diseases, National Institutes of Health, Rockville, Maryland, USA

## Abstract

**Background:**

In sandflies, the blood meal is responsible for the induction of several physiologic processes that culminate in egg development and maturation. During blood feeding, infected sandflies are also able to transmit the parasite Leishmania to a suitable host. Many blood-induced molecules play significant roles during Leishmania development in the sandfly midgut, including parasite killing within the endoperitrophic space. In this work, we randomly sequenced transcripts from three distinct high quality full-length female *Phlebotomus papatasi *midgut-specific cDNA libraries from sugar-fed, blood-fed and *Leishmania major*-infected sandflies. Furthermore, we compared the transcript expression profiles from the three different cDNA libraries by customized bioinformatics analysis and validated these findings by semi-quantitative PCR and real-time PCR.

**Results:**

Transcriptome analysis of 4010 cDNA clones resulted in the identification of the most abundant *P. papatasi *midgut-specific transcripts. The identified molecules included those with putative roles in digestion and peritrophic matrix formation, among others. Moreover, we identified sandfly midgut transcripts that are expressed only after a blood meal, such as microvilli associated-like protein (*PpMVP1*, *PpMVP2 *and *PpMVP3*), a peritrophin (*PpPer1*), trypsin 4 (*PpTryp4*), chymotrypsin *PpChym2*, and two unknown proteins. Of interest, many of these overabundant transcripts such as *PpChym2*, *PpMVP1*, *PpMVP2, PpPer1 *and *PpPer2 *were of lower abundance when the sandfly was given a blood meal in the presence of *L. major*.

**Conclusion:**

This tissue-specific transcriptome analysis provides a comprehensive look at the repertoire of transcripts present in the midgut of the sandfly *P. papatasi*. Furthermore, the customized bioinformatic analysis allowed us to compare and identify the overall transcript abundance from sugar-fed, blood-fed and Leishmania-infected sandflies. The suggested upregulation of specific transcripts in a blood-fed cDNA library were validated by real-time PCR, suggesting that this customized bioinformatic analysis is a powerful and accurate tool useful in analysing expression profiles from different cDNA libraries. Additionally, the findings presented in this work suggest that the Leishmania parasite is modulating key enzymes or proteins in the gut of the sandfly that may be beneficial for its establishment and survival.

## Background

Cutaneous leishmaniasis due to *L. major *is found throughout the Old World, including the Middle East and West Africa. *Phlebotomus papatasi *is the principal vector for this parasite and is refractory to the development of other species of Leishmania.

Upon taking a blood meal, hematophagous arthropods express a large number of molecules that participate in various physiologic processes ranging from blood digestion to egg development. Furthermore, many insects can either obtain or transmit pathogens during the acquisition of a blood meal. In blood-feeding arthropods, the midgut plays a crucial role as the primary organ involved in processing the blood meal and, in some instances, molecules expressed in the midgut of an insect vector have been shown to directly influence pathogen establishment [1,2]. Certain pathogens, such as Leishmania, appear able to modulate the activity of sandfly midgut proteases for their own benefit or survival [3,4].

Sequenced data sets containing information regarding expression profiles of anopheline and culicine mosquitoes, such as *Anopheles gambiae *and *Aedes aegypti*, following a blood meal have become available [5,6]. Other datasets now encompass insects such as *Pedicullus humanus *[7] and *Cullicoides sonorensis *[8]. In comparison, transcriptome information regarding sandflies is limited. Previous work has focused mainly on the sandfly salivary gland [9-11], whereas only a small number of sandfly-specific midgut cDNA have been identified [12-16]. Recently, a large set of cDNA transcripts from the whole sandfly *Lutzomyia longipalpis *has been sequenced, providing greater information regarding molecules present in sandflies [17]. However, the information regarding sandfly midgut-specific transcripts remains poor.

In this work, we embarked on a comprehensive study of *P. papatasi *midgut-specific transcripts and compared the expression profile of these transcripts by directly comparing those obtained from midguts of females fed on sugar only, on blood or on blood containing *L. major*. With this approach, we have identified several *P. papatasi *midgut-specific transcripts that are differentially expressed after a blood meal and in the presence of *L. major*.

## Results and discussion

The midgut is the tissue where Leishmania development takes place while within its sand fly vector. Within the midgut environment, Leishmania possibly interacts with various secreted molecules and cell types lining the midgut epithelia. In order to gain greater insight into the repertoire of the proteins present in the midgut of *P. papatasi*, we constructed and sequenced three high quality full-length cDNA libraries from the midgut of sandflies fed either on sugar only (unfed), blood or blood containing *L. major*. 4010 high quality sequenced clones obtained from the three cDNA libraries were combined and analysed resulting in the formation of 1382 clusters. Each cluster may contain a large number of transcripts which creates a contig (high quality consensus sequence) or may have a single transcript that can be defined as a singleton. Therefore, we will utilise the nomenclature of "cluster" in the remainder of the manuscript to define either a consensus sequence from various transcripts or a singleton.

Consensus sequences were compared with various databases and putative functions were assigned. The categories for the transcripts' potential biologic functions included protein synthesis machinery, protein modification machinery, transcription machinery, transporters, extracellular matrix, signal transduction, immunity, adhesion, and conserved proteins of unknown function. Table 1 summarizes this analysis listing transcripts from female *P. papatasi *midguts fed on sugar, on blood, and on blood containing *L. major*. The first column shows the putative biological function, the first section of columns shows the number of clusters found in each of the three cDNA libraries in relation to this function; the second section of columns indicates the total number of sequences for these clusters and the third section of columns shows the average of the number of sequences per cluster. The category of "conserved unknown function" had the largest number of clusters in all three of the cDNA libraries. These were followed by metabolism, energy in the sugar-fed library (95 clusters); metabolism, amino acid, which includes digestive enzymes, in the blood meal library (40 clusters); and protein synthesis machinery in the *L. major *blood-meal library (51 clusters). The categories with the highest number of sequences per cluster differed between the three cDNA libraries and was highest among transcripts identified as extracellular matrix (27.33 seq/cluster) in the sugar-fed cDNA library and cytoskeletal transcripts for both the blood meal (19.40 seq/cluster) and *L. major *blood meal cDNA libraries (15.00 seq/cluster). The sugar-fed cDNA library has 669 clusters with an average of 3.23 sequences per cluster. The cDNA library constructed from blood-fed midguts consisted of 441 clusters with an average of 3.27 sequences per cluster. Of *P. papatasi *midgut fed on blood containing *L. major*, this library produced 555 clusters with an average of 3.01 sequences per cluster.

**Table 1:** List of *Phlebotomus papatasi *midgut-specific sequences, clusters, and sequences per cluster of cDNA libraries made from flies sugar-fed, blood-fed, and blood fed with *Leishmania major *parasites

	**Number of Clusters**			**Number of Sequences**			**Sequences/cluster**		
**Biological function**	**Sugar**	**Blood-fed**	***L. major***	**Sugar**	**Blood-fed**	***L. major***	**Sugar**	**Blood-fed**	***L. major***

protein synthesis machinery	88	39	51	281	73	159	3.19	1.87	3.12
protein modification machinery	19	20	8	39	22	9	2.05	1.10	1.13
protein export machinery	14	13	10	14	13	10	1.00	1.00	1.00
transcription machinery	4	2	1	4	2	1	1.00	1.00	1.00
transcription factors	13	6	3	22	9	4	1.69	1.50	1.33
proteasome machinery	13	7	7	21	7	9	1.62	1.00	1.29
transporters	24	16	11	37	19	33	1.54	1.19	3.00
extracellular matrix	6	12	5	164	122	59	27.33	10.17	11.80
cytoskeletal	16	15	14	61	291	210	3.81	19.40	15.00
signal transduction	28	20	13	38	20	15	1.36	1.00	1.15
protease inhibitor	3	1	3	8	5	9	2.67	5.00	3.00
immunity	3	4	3	8	5	4	2.67	1.25	1.33
adhesion	1	2	1	1	2	1	1.00	1.00	1.00
nuclear metabolism and regulation	17	7	6	18	9	6	1.06	1.29	1.00
metabolism, energy	95	38	42	194	54	62	2.04	1.42	1.48
metabolism, lipid	9	11	9	11	18	10	1.22	1.64	1.11
metabolism, carbohydrate	16	9	10	27	12	14	1.69	1.33	1.40
metabolism, amino acid	28	40	30	131	182	197	4.68	4.55	6.57
metabolism, nucleic acid and nucleotides	7	9	4	14	9	4	2.00	1.00	1.00
metabolism, heme	3	3	2	8	28	8	2.67	9.33	4.00
conserved of unknown function	262	167	322	405	282	470	1.55	1.69	1.46

**Total**	669	441	555	1506	1184	1294	**Avg **3.23	**Avg **3.27	**Avg **3.01

**Figure 1:**
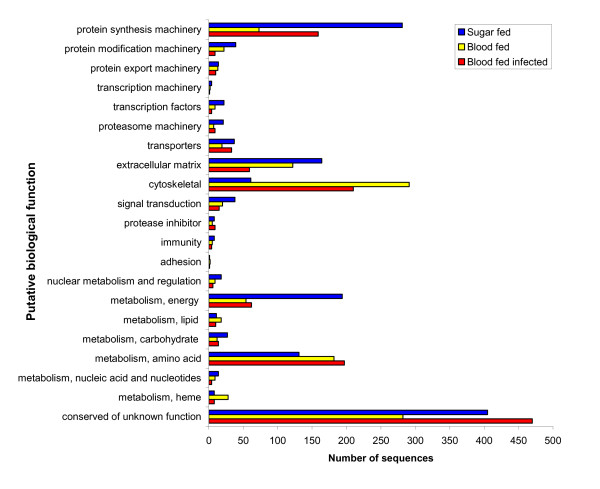
Distribution of sequences analysed from each cDNA library separated by putative biologic function.

The number of sequences in each category for the three cDNA libraries is graphically represented in Figure 1. After blood feeding, there is a decrease in the number of sequences in all categories other than cytoskeletal, amino acid metabolism, and heme metabolism. Noticeable differences in the number of sequences between the blood-fed and blood-fed containing *L. major *libraries occurs in the protein synthesis machinery, extracellular matrix, cytoskeletal, heme metabolism, and conserved of unknown function categories.

Table 2 gives a more detailed description of the different types of transcripts identified in the combined analysis of the three cDNA libraries. Only high quality sequences and, for the most part, full-length coding sequences submitted to GenBank are shown. This table shows the different clusters arranged in the order of cluster number in the combined analysis of the three cDNA libraries. The first column of Table 2 describes the cluster number, the second column shows the clone that produced the full-length sequence, the third column shows the best match in the non-redundant protein database (GenBank, NCBI), the fourth column shows the e-value for the best matching BLAST result in column 3, the fifth column shows the assigned putative function of that cluster, and the sixth column shows the accession number of the transcript submitted to GenBank. The four most abundant transcripts were microvilli-associated like protein, followed by peritrophin-like protein, 40 S ribosomal protein S30 and a transcript coding for a protein of unknown function. Still, other abundant transcripts include those coding for various ribosomal proteins, chymotrypsins, carboxypeptidases, trypsins, a zinc metalloprotease astacin, a Kazal-type serine protease inhibitor, Glutathione S-transferase (GST) and various proteins of unknown function (Table 2). All the sequences generated from these three cDNA libraries have been deposited as an EST database at the National Center of Biological Information (NCBI), accession numbers ES346912 – ES351350 and ES351429). The following is a more detailed description of relevant transcripts identified in the cDNA libraries:

**Table 2:** Clusters of combined *P. papatasi *midgut cDNA libraries (sugar-fed, blood-fed and *Leishmania major *-infected) of transcripts with high quality sequences

**Cluster**	**Clone**	**NCBI best match to NR database**	**E value**	**Putative function**	**GenBank**
Cluster 1	B05_ppmgbl_p23	microvilli membrane protein [*A. aegypti *]	1.0E-47	Microvilli protein	EU031911
Cluster 2	F05_ppmgbl_p23	microvilli membrane protein *[A. aegypti *]	1.0E-47	Microvilli protein	EU031911
Cluster 3	PPMGBM189	microvilli membrane protein [A. aegypti]	7.0E-48	Microvilli protein	EU031911
Cluster 9	PPMGBL17	LP07759p [*D. melanogaster *]	8.0E-25	Peritrophin	EU031912
Cluster 10	A05_PPMGBS_P28	hypothetical protein 17 [*L. obliqua *]	3.0E-44	40S ribosomal S30 protein	EU040041
Cluster 11	A07_PPMGBS_P28	ENSANGP00000028746 [*A. gambiae *]	1.0E-33	Unknown	EU040042
Cluster 12	PPMGM173	similar to CG4778-PA [*T. castaneum *]	8.0E-11	Peritrophin	EU047543
Cluster 13	A02_PPINFL_P30	similar to CG4778-PA [*T. castaneum *]	7.0E-11	Peritrophin	EU047543
Cluster 15	H08_PPMGBL_P31	chymotrypsin [P. papatasi]	1.0E-154	Chymotrypsin	AAM96939
Cluster 16	E10_ppmgbm_p21	carboxypeptidase B [*A. aegypti *]	4.0E-67	Carboxypeptidase	EU047544
Cluster 17	C07_PPMGS_P24	ribosomal protein S20 [*B. mori *]	9.0E-57	40S ribosomal protein S20	EU047545
Cluster 18	H10_ppmgm_p22	trypsin 1 [*P. papatasi *]	1.0E-151	Trypsin	AAM96940
Cluster 20	H05_ppmgm_p22	CG32276-PB, isoform B [*D. melanogaster *]	2.0E-23	Ribosome associated membrane protein	EU047546
Cluster 21	PPMGBS47	Ribosomal protein L19 [*D. melanogaster *]	1.0E-100	60s ribosomal protein L19	EU047547
Cluster 23	D05_PPMGL_P28	trypsin 2 [*P. papatasi *]	1.0E-157	Trypsin	AAM96941
Cluster 24	G04_PPMGM_P25	RE59709p [*D. melanogaster *]	7.0E-68	60S ribosomal protein L32	EU047548
Cluster 25	PPMGS_P31_B06	similar to *D. melanogaster *CG3203 [*D. yakuba *]	1.0E-91	60S ribosomal protein L17	EU045355
Cluster 26	PPMGL_P29_D06	peritrophin-like protein 1 [*C. felis *]	2.0E-36	Peritrophin	EU045354
Cluster 29	PPINFM-P7-G10	Ribosomal protein L29 [*D. melanogaster *]	4.0E-24	60S ribosomal protein L29	EU045353
Cluster 31	B03_PPMGM_P25	CG13551 [*D. melanogaster *]	3.0E-37	Unknown	EU049582
Cluster 32	H04_PPMGL_P23	LD17235p [*D. melanogaster *]	1.0E-93	60S ribosomal protein L11	EU045352
Cluster 34	PPMGM152	similar to *D. melanogaster *RpL14 [*D. yakuba *]	5.0E-50	60S ribosomal protein L14	EU045351
Cluster 35	E07_ppmgs_p21	60S acidic ribosomal protein P1 [*S. frugiperda *]	1.0E-45	60s Acidic ribosomal protein P1	EU045350
Cluster 37	PPMGL197	ENSANGP00000019623 [*A. gambiae *]	4.0E-63	Astacin	EU045349
Cluster 40	F01_PPINFL_P30	S7 ribosomal protein [*C. pipiens quinquefasciatus *]	3.0E-89	40S ribosomal protein S7	EU045348
Cluster 73	A03_PPPMGBM_P25	unknown [*C. sonorensis *]	4.0E-25	Unknown	EU045347
Cluster 75	C04_ppmgs_p21	similar to *D. melanogaster *qm [*D. yakuba *]	1.0E-117	60s ribosomal protein L10	EU045346
Cluster 89	B11_PPINFL_P32	trypsin 4 [*P. papatasi *]	1.0E-129	Trypsin	AAM96943
Cluster 94	F08_PPINFM_P22	microvilli membrane protein [*A. aegypti *]	2.0E-35	Microvilli protein	EU047549
Cluster 96	C05_ppmgm_p22	Cr-PII [*P. americana *]	3.0E-20	Microvilli protein	EU047550
Cluster 98	B12_ppmgbl_p23	ENSANGP00000017713 [*A. gambiae *]	1.0E-17	Microvilli protein	EU047551
Cluster 99	A03_ppmgbl_p20	hypothetical protein [*T. castaneum *]	2.0E-23	Unknown	EU045345
Cluster 103	H03_PPMGL_P23	GA13179-PA [*D. pseudoobscura *]	2.0E-43	Ferritin	EU045344
Cluster 106	H07_ppmgm_p22	TPA_inf: HDC07203 [*D. melanogaster *]	8.0E-14	Unknown	EU045343
Cluster 111	PPMGL_P34_H09	GA16408-PA [*D. pseudoobscura *]	2.0E-10	Kazal type serine protease inhibitor	EU045342
Cluster 113	B10_PPINFL_P21	carboxypeptidase A [*A. aegypti *]	2.0E-87	Carboxypeptidase	EU045341
Cluster 119	PPMGL_P29_B04	midgut specific galectin [*P. papatasi *]	1.0E-145	Galectin	AAT11557
Cluster 122	C08_PPINFL_P31	GA15307-PA [*D. pseudoobscura *]	3.0E-62	Ferritin	EU045340
Cluster 125	D06_PPMGL_P23	Glutathione S-transferase [M. domestica]	3.0E-86	Glutathione S-transferase	EU045339
Cluster 126	PPMGBL175	10 kDa salivary protein [*P. ariasi *]	6.0E-07	Unknown	EU045338
Cluster 127	H07_ppmgs_p21	ribosomal protein S8 [*A. albopictus *]	3.0E-94	40S ribosomal protein S8	EU045337
Cluster 128	C02_ppmgs_p21	60S acidic ribosomal protein P2 [*A. aegypti *]	8.0E-39	60S acidic ribosomal protein P2	EU045336
Cluster 129	PPMGL_P29_C01	ENSANGP00000016569 [*A. gambiae *]	5.0E-40	membrane LPS inducible TNF protein	EU035828
Cluster 134	C01_PPINFM_P22	similar to *D. melanogaster *RpS18 [*D. yakuba *]	9.0E-70	40S ribosomal protein S18	EU035827
Cluster 135	D04_PPINFL_P31	trypsin 3 [*P. papatasi *]	1.0E-144	Trypsin	AAM96942
Cluster 139	PPMGS_P31_A11	CG30415-PB, isoform B [*D. melanogaster *]	1.0E-27	Unkown	EU035823
Cluster 146	PPMGM132	similar to CG2998-PA [*T. castaneum *]	2.0E-27	40S ribosomal protein S28	EU035822
Cluster 147	A06_PPMGM_P25	Ribosomal protein L23 [*D. melanogaster *]	5.0E-73	60S ribosomal protein L23	EU035821
Cluster 149	B06_PPMGM_P25	similar to *D. melanogaster *RpS12 [*D. yakuba *]	1.0E-64	40S ribosomal protein S12	EU035820
Cluster 150	PPMGL_P29_D08	ENSANGP00000021011 [*A. gambiae *]	1.0E-111	Unkown	EU035819
Cluster 153	B07_PPMGS_P24	similar to *D. melanogaster *CG2033 [*D. yakuba *]	3.0E-67	40S ribosomal protein S15	EU035818
Cluster 158	D06_PPINFL_P21	ENSANGP00000013724 [*A. gambiae *]	5.0E-70	Ryanodine receptor	EU035817
Cluster 163	PPMGM133	cyclophylin isoform [*A. aegypti *]	8.0E-82	Cyclophilin	EU032351
Cluster 165	A11_PPMGM_P25	60S ribosomal protein L40 [*A. albopictus *]	4.0E-68	Ubiquitin/ribosomal L40 fusion	EU032350
Cluster 167	PPMGL276	similar to ENSANGP00000002356 [*A. mellifera *]	2.0E-66	Na+/K+ ATPase	EU032348
Cluster 168	F05_ppmgbm_p21	chymotrypsin [*P. papatasi *]	1.0E-147	Chymotrypsin	AAM96938
Cluster 171	G12_ppmgs_p21	GA16582-PA [*D. pseudoobscura *]	8.0E-78	60S ribosomal protein L12	EU032349
Cluster 174	A06_ppmgm_p22	similar to *D. melanogaster *CG2099 [*D. yakuba *]	1.0E-54	60S ribosomal protein L35A	EU032347
Cluster 176	A03_pppmgbl_p24	translation factor SUI1-like protein [*A. aegypti *]	1.0E-54	Translation initiation factor 1	EU032346
Cluster 177	A08_PPMGBS_P28	GA10714-PA [*D. pseudoobscura *]	5.0E-92	ADP ribosylation factor	EU032345
Cluster 182	PPMGS_P31_E04	similar to *D. melanogaster *CG10423 [*D. yakuba *]	2.0E-38	40s ribosomal protein S27	EU032344
Cluster 183	D12_ppmgm_p22	ENSANGP00000026718 [*A. gambiae *]	1.0E-61	Cytochrome C oxidase subunit IV	EU032343
Cluster 184	C02_PPINFL_P30	unknown [*C. sonorensis *]	4.0E-21	Unknown	EU032342
Cluster 185	D04_PPINFM_P27	cytochrome b [*P. papatasi *]	1.0E-110	Cytochrome B	AF161214
Cluster 186	MGL69	similar to CG9916-PA isoform 1 [*T. castaneum *]	4.0E-81	Cyclophilin	EU032341
Cluster 187	PPINFM-P7-F11	ribosomal protein S17e [*Eucinetus sp*. ]	9.0E-31	40S ribosomal protein S17	EU032340
Cluster 188	H01_PPMGL_P23	10 kDa salivary protein [*P. ariasi *]	2.0E-06	Unknown	EU032339
Cluster 201	G11_PPPMBGM_P26	larval chymotrypsin-like protein [*A. aegypti *]	5.0E-82	Chymotrypsin	EU035826
Cluster 228	PPMGM508	peroxiredoxin-like protein [*A. aegypti *]	1.0E-69	Peroxiredoxin	EU035825
Cluster 232	PPMGBL92	glutathione S-transferase [*A. aegypti *]	4.0E-59	Glutathione S-transferase	EU035824
Cluster 243	D11_pppmgbl_p24	midgut chitinase [*P. papatasi *]	1.0E-140	chitinase	AAV49322

### Microvilli-associated like proteins

**Figure 2:**
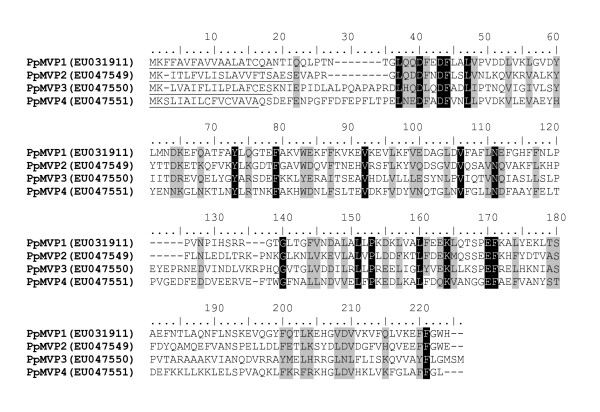
Multiple sequence alignment of the four putative microvilli associated-like proteins found in the midgut of *Phlebotomus papatasi*. Predicted signal peptide sequence is underlined and the accession numbers given in parentheses.

Of the most abundant transcripts found in the combined analysis of all three libraries were transcripts coding for proteins with similarities to microvilli membrane proteins from *A. aegypti *and *A. gambiae*. These transcripts are also homologous to major allergens identified in the cockroaches *Blatella germanica *and *Periplaneta Americana *[18] and to a nitrile-specifier protein (PrNSP) from the midgut of *Pieris rapae*. PrNSP has a role of converting toxic compounds, such as isothiocyanate, into less toxic compounds, such as nitriles, that are excreted in the feces of larval stages of this lepidopteran [19]. Four different putative microvilli-associated proteins were identified in the three *P. papatasi *midgut cDNA libraries (Figure 2). Clusters 1, 2, and 3 represent likely polymorphisms of the same transcript named here "microvilli protein 1" (*PpMVP1*), which has a predicted molecular weight of 23.7 kDa. Another three transcripts coding for microvilli proteins and derived from clusters 94, 96 and 98 were named *PpMVP2*, *PpMVP3*, and *PpMVP4*, respectively. The predicted molecular weight for these microvilli-associated like proteins is 24.0, 25.6, and 25.6 kDa, respectively. Additionally, each of these microvilli proteins has a potential signal peptide as predicted by SignalP 3.0 and no evidence of transmembrane helices as predicted using the TMHMM 2.0 server. Identity between the amino acid sequences of these microvilli proteins ranges from 21 to 36 percent (Figure 2, black-shaded amino acids) and similarity from 45 to 57 percent (Figure 2, grey-shaded amino acids). The degree of conservation may indicate that these are biochemically distinct from one another and only commonly named based on the previous annotation of other organisms with similar sequences. Searching the translated assembled sequences from an EST database of *L. longipalpis *identified NSFM-139c08, NSFM-18h11, NSFM-68e08, and NSFM-47h07 as having high sequence homology to the microvilli-associated like proteins *PpMVP1*, *PpMVP2*, *PpMVP3*, and *PpMVP4*, respectively [17].

### Peritrophin-like proteins

**Figure 3:**
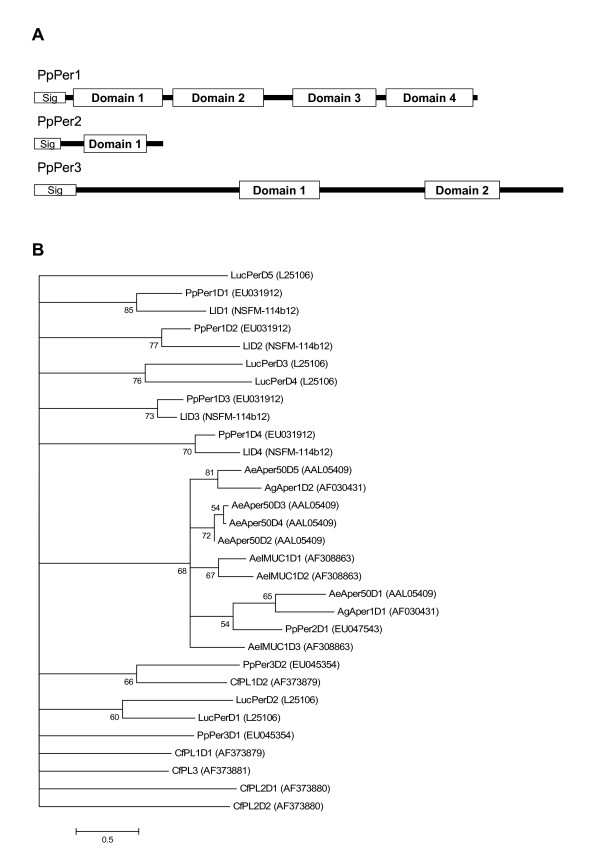
Characterisation of peritrophin sequences. (A) Diagrammatic representation of *Phlebotomus papatasi *peritrophin-like molecules showing the predicted signal peptide and chitin-binding domains. (B) Phylogenetic analysis of chitin-binding domains of peritrophin molecules from *Aedes aegypti *(Ae), *Anopheles gambiae *(Ag) *Ctenocephalides felis *(Cf), *Lucilia cuprina *(Luc), *Phlebotomus papatasi *(Pp), *Lutzomyia longipalpis *(Ll). Accession numbers are indicated in parenthesis and bootstrap values at the nodes.

Transcripts coding for three different putative peritrophin-like molecules were identified in the midgut of *P. papatasi*. *PpPer1 *(cluster 9) and *PpPer2 *(clusters 12 and 13) transcripts code for secreted proteins with predicted molecular masses of 29.8 and 9.6 kDa, respectively. PpPer1 is comprised of four potential chitin-binding peritrophin-A domains (Figure 3A). PpPer2 is a much smaller predicted protein and has only one potential chitin-binding domain (Figure 3A). A third putative peritrophin, *PpPer3*, was identified from cluster 26 with an apparent molecular mass of approximately 32 kDa (Figure 3A) and contains two distant putative chitin-binding domains. Phylogenetic analysis using the chitin binding domains of PpPer1, Pper2, PpPer3 and those of peritrophins from several insects (Figure 3B) suggests a low level of conservation between the domains. Insect peritrophins have been reported to bind to chitin fibers via multiple chitin-binding domains, forming the scaffold that maintains the molecular structure of the peritrophic matrix (PM) in the insect gut [20]. In addition to their role in the formation of the PM, peritrophins may also play a role in preventing the toxic effects of heme, a bi-product of blood meal digestion. In *A. aegypti*, AeIMUC1, a mucin that encodes putative chitin-binding domains was recently shown to bind heme [21]. Although peritrophins have been characterised from several insects, including *A. aegypti *and *A. gambiae *[20,22], no information exists related to sandfly midgut-specific peritrophins. PpPer1 and PpPer3 have high sequence similarity, at the protein level, to the translated sequences SFM-03c06 and SFM-02h07 from the *L. longipalpis *EST database. However, PpPer2 has lower sequence similarity to any of the assembled and translated sequences from the *L. longipalpis *EST database, suggesting a more divergent or novel molecule.

### Trypsins

**Figure 4:**
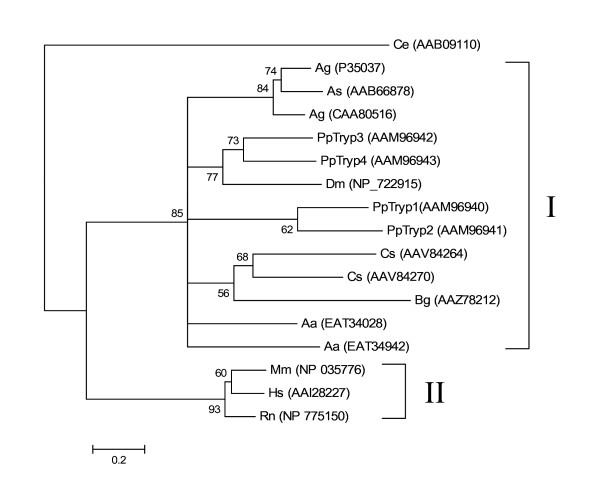
Phylogenetic analysis of trypsins from *Caenorhabditis elegans *(Ce), *Rattus norvegicus *(Rn), *Mus musculus *(Mm), *Homo sapiens *(Hs), *Blattella germanica *(Bg), *Anopheles gambiae *(Ag), *Anopheles stephensi *(As), *Aedes aegypti *(Aa), *Drosophila melanogaster *(Dm), *Culicoides sonorensis *(Cs), and *Phlebotomus papatasi *(Pp). The accession number of the sequence used is in parentheses and node support indicated by the bootstrap values.

Among the most abundant transcripts in the cDNA libraries were the previously characterised *P. papatasi *trypsin-like, *PpTryp1*, (Cluster 18 with 158 sequences), and *PpTryp4 *(Cluster 89 with 114 sequences) [13]. *PpTryp2 *(Cluster 23)and *PpTryp3 *(Cluster 135) were less abundant with 12 and 8 sequences, respectively. Phylogenetic analysis of trypsins from *P. papatasi *and from other organisms resulted in the formation of two major clades (Figure 4). *P. papatasi *trypsins co-localised in clade I containing other insect trypsins, while their mammalian counterparts were found in clade II (Figure 4). As detected previously, [13] PpTryp1 and PpTryp2 form a different clade apart from a clade formed by PpTryp3 and PpTryp4 (Figure 4). The *P. papatasi *trypsins PpTryp1, PpTryp2, PpTryp3, PpTryp4, show high protein sequence similarity to *L. longipalpis *ESTs NSFM-02a01, NSFM-113h06, NSFM-94b08, and NSFM-165c07, respectively.

### Chymotrypsin

**Figure 5:**
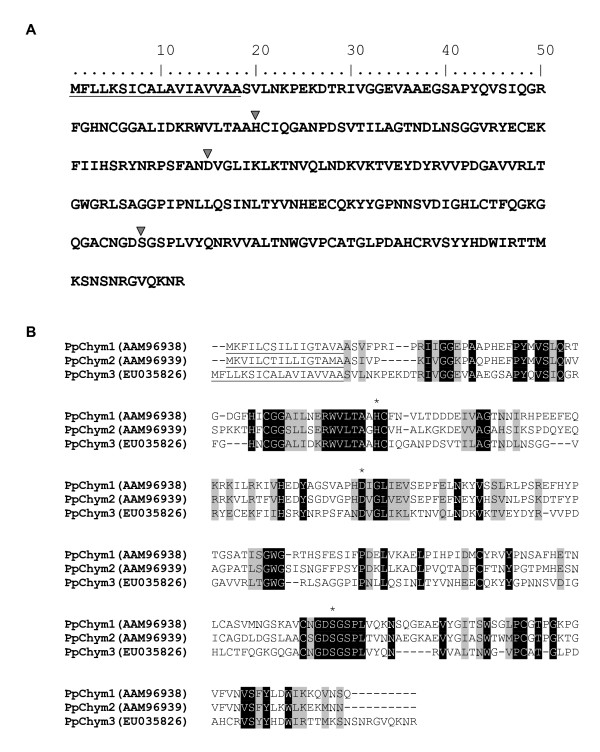
Chymotrypsin sequence analysis. (A) Diagrammatic representation of PpChym3 sequence showing the predicted signal peptide (underlined) and the residues of the catalytic triad (H/D/S) marked with a triangle. (B) Sequence alignment of the three *Phlebotomus papatasi *chymotrypsin-like sequences. Identical residues are highlighted in black and similar residues highlighted in grey. The predicted signal peptides are underlined and the catalytic residues marked with (*) and the accession numbers are in parentheses.

Two previously characterised *P. papatasi *chymotrypsin-like cDNA, *PpChym1 *and *PpChym2 *[13], as well as a novel chymotrypsin-like, *PpChym3 *(Cluster 113) were also found in the transcriptome database. This newly identified novel chymotrypsin-like molecule was found in low abundance in the blood-fed midgut library. The predicted Ppchym3 has 36% amino acid identity to Ppchym1 and 30% amino acid identity to Ppchym2. Furthermore, Ppchym3 has a signal secretory peptide (Figure 5A) and has the required His/Asp/Ser amino acid triad necessary for catalytic activity (Figure 5B). Ppchym1 and Ppchym2 both share sequence homology from the assembled sequence NSFM-01d03 from the *L. longipalpis *EST database, while Ppchym3 is most similar to sequence SFM-01b03.

### Carboxypeptidase

**Figure 6:**
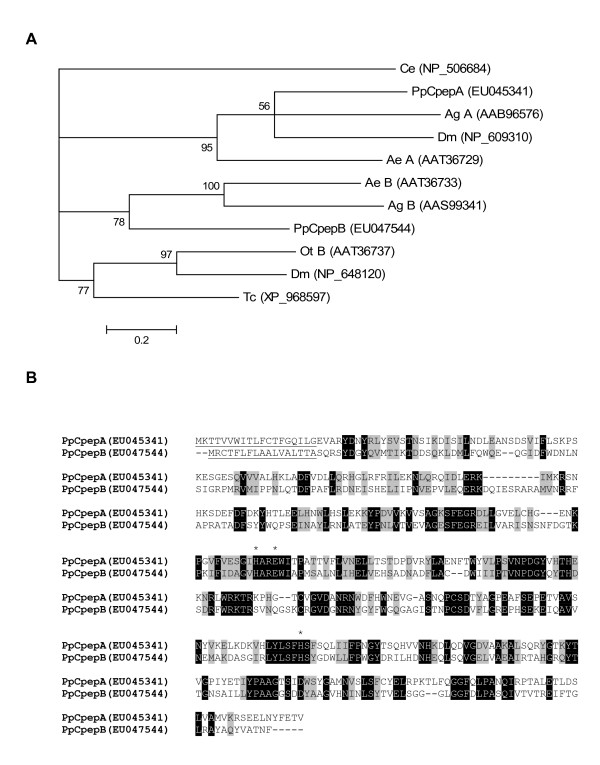
*Phlebotomus papatasi *midgut carboxypeptidase like proteins. (A) Phylogenetic analysis of carboxypeptidases from *Caenorhabditis elegans *(Ce), *Aedes aegypti *(Ae), *Anopheles gambiae *(Ag), *Drosophila melanogaster *(Dm), *Ochlerotatus triseriatus *(Ot), *Tribolium castaneum *(Tc), and *Phlebotomus papatasi *(Pp). Accession numbers are indicated in parenthesis and node support indicated by the bootstrap values. (B) Sequence comparison of midgut *Phlebotomus papatasi *carboxypeptidase A (PpCpepA) and carboxypeptidase B (PpCpepB). The predicted signal peptide is underlined and the residues necessary for zinc binding (H and E) are indicated by (*).

A number of sequences were identified with homology to carboxypeptidases. The full-length transcript of a putative carboxypeptidase B,*PpCpepB*, was found from 37 sequences in cluster 16 and has high homology to a carboxypeptidase B identified in *A. aegypti *(GenBank accession# AAT36733). The predicted amino acid sequence of PpCpepB contains a signal peptide, a propeptide domain, and a carboxypeptidase domain. A putative carboxypeptidase A, *PpCpepA*, was also identified from cluster 113 based on amino acid sequence homology. Phylogenetic analysis shows that the identified *P. papatasi *putative carboxypeptidases are separated into distinct clades (Figure 6A). Comparison of sequence homology indicates the potential for these molecules to have substrate specificities of either carboxypeptidases A or B (Figure 6B). Sequence alignment of the two carboxypeptidases depicts the difference in amino acid composition; however, both sequences contain the zinc ion binding motifs of metallocarboxypeptidases (Figure 6B). Additionally, the presence of a putative signal peptide alludes that these molecules are midgut digestive enzymes. Similarity between these carboxypeptidases and those present in *L. longipalpis *EST database is evident by the high homology between PpCpepA and SFM-05c11 and between PpCpepB and NSFM-32d09.

### Astacin-like zinc metalloprotease

**Figure 7:**
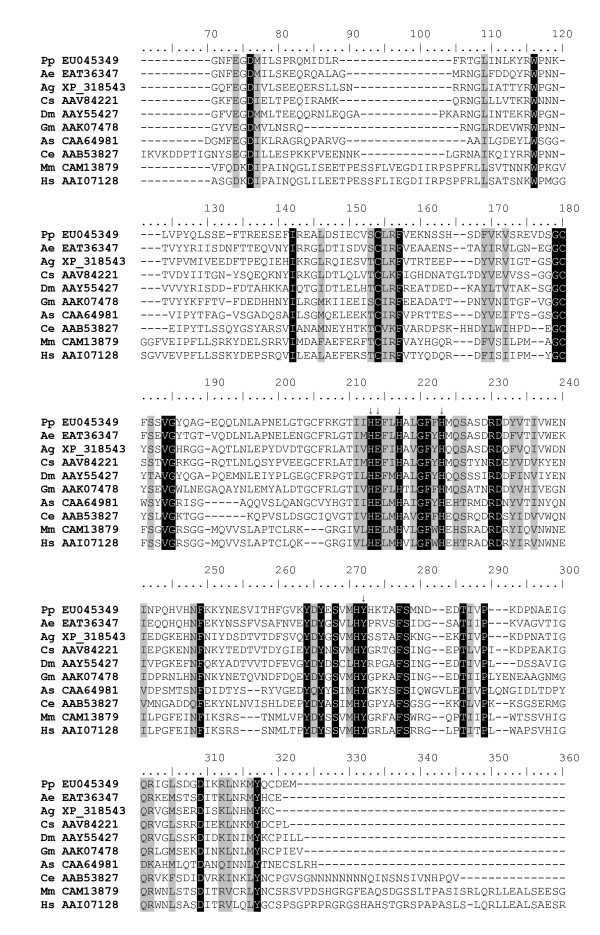
Multiple sequence analysis of astacin-like proteins. Sequence alignment of zinc proteases astacin-like sequences from *Phlebotomus papatasi *(Pp), *Aedes aegypti *(Ae), *Anopheles gambiae *(Ag), *Culicoides sonorensis *(Cs), *Drosophila melanogaster *(Dm), *Glossina morsitans morsitans *(Gm), *Astacus astacus *(As), *Caenorhabditis elegans *(Ce), *Mus musculus *(Mm), and *Homo sapiens *(Hs). Arrows indicate the residues likely necessary for catalytic activity. Accession numbers are shown.

A putative astacin-like zinc metalloprotease (*PpAstacin*) was identified from cluster 37, a product of five sequences. This putative astacin-like protein displays a predicted signal peptide and a slightly modified form of the signature zinc binding catalytic domain for proteins in the astacin family (HEXXHXXGFXHEXXRXDR). In PpAstacin, changes in two residues (E to M and R to A) resulted in the motif HEFLHALGFFHMQSASDR (Figure 7A). Although the altered residues may be involved in target specificity the zinc-binding catalytic domain remains conserved. The likely role of this putative protein is blood meal digestion, as astacins molecules have not been implicated in immune functions and a considerable number of transcripts constituting this cluster were derived from the blood-fed midgut cDNA library. This is the first report of this type of protease from the gut of a sandfly, though NSFM-127b08 of the *L. longipalpis *EST database was identified based on sequence homology.

### Kazal-type serine protease inhibitor

**Figure 8:**
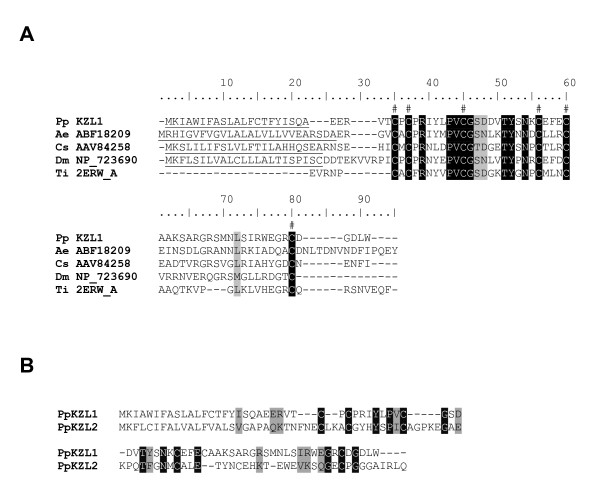
Sequence analysis of Kazal-type proteins. (A) Sequence alignment of Kazal-type proteins from *Phlebotomus papatasi *(Pp), *Aedes aegypti *(Ae), *Culicoides sonorensis *(Cs), *Drosophila melanogaster *(Dm) and *Triatoma infestans *(Ti). The predictedsignal peptide sequences are undelined and the conserved cysteineresidues denoted by #. Identical residues are highlighted in blackand similar residues highlighted in grey. PpKZL1 accession number is EU045342 (B) Sequence comparison of the two Kazal-type proteins(PpKZL1 and PpKZL2) from *Phlebotomus papatasi *found in themidgut cDNA libraries. Identical residues are highlighted in blackand similar residues highlighted in grey.

Two Kazal-type serine protease inhibitors were identified from cluster 111 (*PpKZL1*) and 859 (*PpKZL2*) in the cDNA midgut libraries. *PpKZL1 *codes for a small peptide of 78 amino acids while *PpKZL2 *codes for a peptide of 89 amino acids. Both proteins are predicted to be secreted based on the presence of signal peptides (Figure 8). PpKZL1 is similar to various small Kazal-type inhibitors found in *Drosophila pseudoobscura *(gi: 125986397), *C. sonorensis *(gi:56199538) and the mosquitoes *A. aegypti *and *A. gambiae*, and to larger Kazal-type molecules such as infestin [23] from *Triatoma infestans *(Figure 8A). There is only 28% identity and 42 % similarity between PpKZL1 and PpKZL2 (Figure 8B) suggesting these may have different functions. Additionally, these two Kazal-type cDNAs are similar to the previously characterised thrombin inhibitor, rhodniin,, from the triatomine *Rhodnius prolixus *[24] (data not shown). Due to their anti-hemostatic effect, rhodniin and infestin are believed to play a role in the fluidity of the blood within the midgut of these vectors. It is conceivable that one or both transcripts coding for Kazal-type thrombin inhibitors identified in *P. papatasi *may play a role in blood fluidity within the sandfly midgut, allowing it to be fully digested by the various proteases secreted within the midgut following the blood meal. These represent the first Kazal-type serine protease inhibitors identified from sandflies. PpKZL2 shares low sequence similarity with SFM-0406 from the *L. longipalpis *EST database and no significant similarities were identified for PpKZL1.

### Ferritin

**Figure 9:**
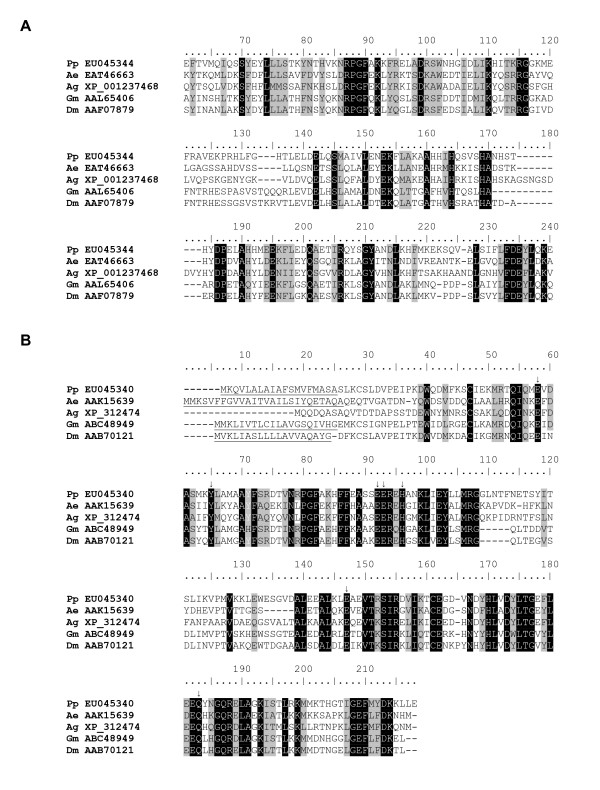
Sequence analysis of ferritin heavy and light chain molecules. Sequence alignment of sequences from *Aedes aegypti *(Ae), *Anopheles gambiae *(Ag), *Glossina morsitans morsitans *(Gm), *Drosophila melanogaster *(Dm), and *Phlebotomus papatasi *(Pp). (A) Light-chain ferritin subunits. (B) Heavy-chain ferritin subunit. Arrows indicate residues associated with the ferroxidase center, the predicted signal peptide sequence is underlined and the accession numbers are given.

Two transcripts encoding putative ferritin light (*PpFLC*) and heavy (*PpFHC*) chain subunits were identified in clusters 103 and 122, respectively (Figure 9). After the ingestion of a blood meal the fly encounters a tremendous dose of iron and heme which would be fatal to most organisms. Ferritin is one of the important factors in controlling the high iron load in hematophagous insects. The midgut of blood-feeding insects envelopes the blood meal and consequently makes the midgut tissue the most likely site of iron regulatory molecules. However, ferritin may also be important for oxidative stress not related to the presence of iron or heme, as it is induced by the presence of H_2_O_2 _in *A. aegypti *[25]. PpFLC and PpFHC are similar to NSFM-144g07 and NSFM-146d09, respectively; molecules identified by searching the *L. longipalpis *EST database.

### Glutathione S-transferase (GST)

From clusters 125 and 232, two transcripts were identified to encode putative GSTs with homology to other dipteran GSTs in the Sigma and Delta/Epsilon classes, respectively. The predicted molecular weights of the two putative proteins are similar at 23.2 kDa for cluster 125 and 24.5 kDa for cluster 232. Within the midgut, these proteins may play an important role in the regulation of reactive oxygen species which occur as a by-product of hemoglobin digestion. Cluster 125 and 232 share high protein sequence similarity with *L. longipalpis *ESTs NSFM-105e10 and NSFM-74c11, respectively.

### Unknown proteins

A large number of clusters produced by the three cDNA libraries have no sequence similarity to other known proteins. This has also been observed in the analysis of the *Chironomus tentans *midgut with good evidence that the unknown transcripts contained coding sequences [26]. It is also possible that the abundance of unidentifiable sequences may be caused by the sequence quality of the transcripts or that the captured sequences are 3' untranslated regions, non-coding small nuclear RNA, or sequences of uncharacterised organisms such as bacteria and yeast present in the sandfly midgut. A number of clusters with unknown functions were identified as coding sequences which exhibited signal peptides, such as clusters 11 and 126.

### Functionally characterised proteins

From the three cDNA libraries, we identified chitinase transcripts which were then expressed as recombinant proteins for the demonstration of activity in the midgut of *P. papatasi *sandflies [15]. Another product of the cDNA libraries was the identification and characterisation of a galectin protein as the first arthropod receptor for a parasite; specifically, *L. major *within the *P. papatasi *sandfly midgut [1].

### Comparative analysis of transcripts that significantly differ from the sugar-fed and blood-fed midgut cDNA libraries

To investigate the effects of blood feeding on the midgut expression profile in *P. papatasi*, we compared the abundance of transcripts in sugar and blood-fed cDNA libraries. We hypothesized that a blood meal will have an effect on the expression of sandfly midgut transcripts that will be reflected in the relative abundance of sequences forming a cluster in the two libraries. Chi-square statistical analysis was used to evaluate the significance of the differences in the abundance of midgut transcripts from unfed and blood-fed cDNA libraries thereby identifying different expression profiles of selected midgut transcripts in each cDNA library.

We observed a significant difference (*P *value ≤ 0.05) in the abundance of a number of midgut transcripts when we compared the sugar-fed and blood-fed sandfly midgut cDNA library. Table 3 shows a list of selected transcripts that were either more abundantly or less abundantly expressed in these two cDNA libraries.

As expected, transcripts coding for proteolytic enzymes such as trypsin (*PpTryp4*), and chymotrypsin (*PpChym2*) were more abundantly represented in the blood-fed cDNA library than in the sugar-fed cDNA library (Table 3). Other transcripts coding for peritrophin and microvilli-like proteins and ferritin were also more abundantly represented in the blood-fed cDNA library. Also, we observed a number of transcripts that were less abundantly represented in the blood-fed cDNA library, such as tryspin 1 (*PpTryp1*), and peritrophin (*PpPer2*).

**Table 3:** Clusters overrepresented in the sugar-fed and blood-fed midgut cDNA libraries as determined by X^2 ^statistical analysis

**Putative function**	**Sugar fed**	**Blood fed**	**P value**	**Genbank**
Microvilli protein (PpMVP1)	0	195	4.3E-58	EU031911
Microvilli protein (PpMVP2)	1	60	1.9E-17	EU047549
Microvilli protein (PpMVP3)	39	8	1.1E-04	EU047550
Microvilli protein (PpMVP4)	0	18	2.4E-06	EU047551
Peritrophin (PpPer1)	0	54	1.9E-16	EU031912
Peritrophin (PpPer2)	152	45	1.1E-10	EU047543
Ferritin light chain (PpFLC)	6	18	2.9E-03	EU045344
Chymotrypsin (Ppchym2)	0	36	2.1E-11	AY128107
Trypsin (PpTryp1)	86	10	4.4E-14	AY128108
Trypsin (PpTryp4)	0	52	6.9E-16	AY128111
Unknown (Cluster 73)	13	21	4.6E-02	EU045347
Unknown (Cluster 99)	0	29	1.9E-09	EU045345

### Validation of transcript abundance of selected sequences by real-time PCR

In order to validate the results observed by the chi-square analysis, we further characterised several transcripts by semi-quantitative end-point reverse-transcriptase PCR as well as by real-time PCR. These were utilised to assess the relative abundance of transcripts in the midgut tissue under sugar-fed and blood-fed conditions. The investigated transcripts included peritrophins *PpPer1 *and *PpPer2*, as well as microvilli proteins *PpMVP1*, *PpMVP2*, and *PpMVP4*.

**Figure 10:**
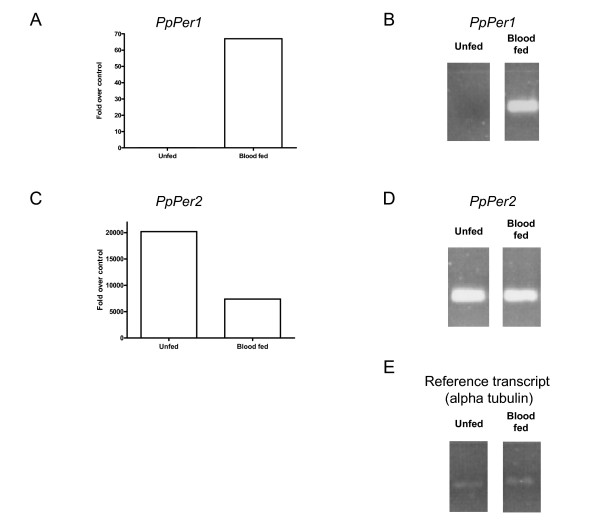
Comparative abundance of peritrophin transcripts in sugar fed or blood fed sand flies. (A, C) *PpPer1 *and *PpPer2 *transcripts fold over control (reference transcript = alpha tubulin) in unfed and blood fed *P. papatasi *midgut. (B, D) Semi-quantitative PCR amplified *PpPer1 *and *PpPer2 *transcripts separated by agarose electrophoresis.

The results of semi-quantitative PCR can be seen in Figures 10B and 10D where the induction of *PpPer1 *is clearly evident. The differences in *PpPer2 *expression between the two midguts conditions is less clear using this technique (Figure 10D). Figure 10A shows the transcript abundance of *PpPer1 *as fold change over the control gene in non blood-fed and post blood-meal ingestion as measured by real-time PCR. Figure 10C shows the same real-time PCR analysis of the *PpPer2 *transcript. The profile of the peritrophin transcripts by real-time PCR strongly correlates with the profile found in the libraries based on the number of sequences.

Based on real-time PCR, *PpPer1 *expression is induced by blood digestion and it is not detected in sugar fed midguts, corresponding with the lack of any sequences produced in the sugar-fed midgut cDNA library, compared to 54 sequences found in the blood fed library. As predicted by the high sequence abundance of *PpPer2 *in the sugar-fed cDNA library the expression of this transcript is highest in unfed sand flies and seems to be down-regulated by the ingestion of a blood meal (Figure 10C).

**Figure 11:**
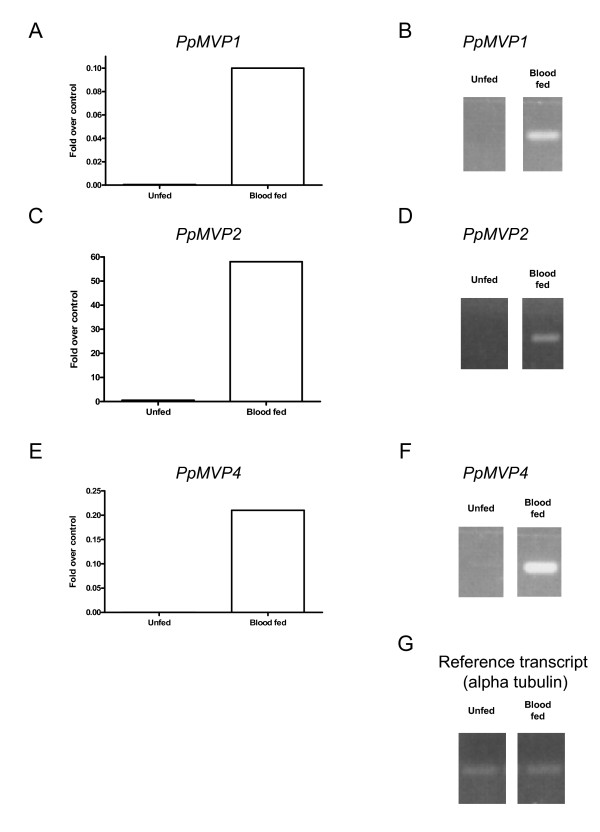
Transcript abundance of microvilli associated-like proteins compared between unfed and blood fed sand flies. A, C, E: *PpMVP1*,*PpMVP2*,*and PpMVP4 *transcript fold over control (reference transcript = alpha tubulin) in unfed and blood fed *P. papatasi *midgut. B, C, F: *PpMVP1*,*PpMVP2*,*and PpMVP4 *semi-quantitative PCR amplified transcripts separated by agarose electrophoresis.

Transcription levels of mRNAs coding for microvilli-like proteins (*PpMVP1*, *PpMVP2*, and *PpMVP4*) tested by semi-quantitative PCR and real-time PCR are shown in Figure 11 and illustrate the induction of transcription by the ingestion of a blood meal. This mirrors what is seen by the sequence abundance of the cDNA library, in which only one sequence of *PpMVP2 *was observed in the sugar-fed cDNA library. The remaining sequences were contributed by the cDNA library produced from blood-fed sandflies.

*Pptryp1 *low and *Pptryp4 *high transcript abundance, were in accordance with the results of previously published endpoint reverse-transcriptase PCR [13]. Additionally, the previously characterised chitinase molecule, *PpChit1*, was identified in cluster 243 and produced by three sequences contributed by the blood-fed cDNA library with none present in the sugar-fed cDNA library. The mRNA expression levels of *PpChit1 *peak at 72 hours post blood-meal ingestion [15].

### Comparative analysis of transcripts significantly differs from the blood-fed and *L. major*-infected midgut cDNA libraries

During its development within the midgut of the sandfly, Leishmania is faced with various potential barriers that may prevent the establishment of the infection. Among such potential barriers are digestive proteases (trypsins and chymotrypsins), the peritrophic matrix and the requirement for parasite attachment to the midgut epithelia to prevent excretion of parasites with remnants of the digested blood. Previous data suggested that Leishmania is able to downregulate proteolytic activity in the sandfly midgut [4]. Also, chitinases produced either by the sandfly [15] or by the Leishmania [27] facilitates parasites in the escape from the peritrophic matrix. Attachment to the midgut epithelia occurs via the presence of *L. major *lipophosphoglycan receptors, such as PpGalec [1] or, in the case of permissive sandflies, via the presence of midgut glycoproteins bearing terminal N-acetyl-galactosamine [28].

In sandflies, only a handful of midgut proteins have been clearly implicated in Leishmania development. Previous data indicated that Leishmania is able to manipulate the activity of certain digestive proteases, inhibiting or delaying their peak activity, possibly in order to survive the proteolytic attack it faces in the midgut of the vector [3,27]. We hypothesized that a blood meal containing *L. major *will affect the expression profile of midgut transcripts altering the abundance of the different transcripts in each of these cDNA libraries. Table 4 shows the results of the chi-square analysis when transcripts from the blood-fed and *L. major*-infected blood-fed cDNA libraries were compared. Of interest, the abundance of transcripts coding for proteolytic enzymes were dramatically decreased in the midgut cDNA library of sandflies fed on *L. major*-infected blood. Additionally, other transcripts that also appear to have their number reduced included those coding for microvilli-associated like proteins and peritrophins. Transcripts such as the one corresponding to *PpTryp1 *(trypsin 1) and one corresponding to *PpPer2 *(peritrophin 2) were more abundant. Other transcripts coding for unknown proteins were also less abundant in the *L. major*-infected cDNA library than in the blood-fed cDNA library. These data suggest that the parasite may be affecting the expression profile of these transcripts and this inhibition, particularly of proteolytic enzymes, may be advantageous for the survival and establishment of the parasite in the midgut of the sandfly.

**Table 4:** Clusters overrepresented in the blood-fed and *Leishmania major*-infected sand fly midgut cDNA libraries as determined by X^2 ^statistical analysis

**Putative function**	**Blood fed**	***L. major***	**P value**	**Genbank**
Microvilli protein (PpMVP1)	134	70	5.8E-07	EU031911
Microvilli protein (PpMVP2)	60	42	4.1E-02	EU047549
Peritrophin (PpPer1)	54	16	1.7E-06	EU031912
Peritrophin (PpPer2)	45	35	1.8E-02	EU047543
Ferritin light chain (PpFLC)	18	3	7.1E-04	EU045344
Chymotrypsin (Ppchym2)	36	8	1.1E-05	AY128107
Trypsin (PpTryp1)	10	82	1.0E-13	AY128108
Unknown (Cluster 73)	21	6	2.6E-03	EU045347
Unknown (Cluster 99)	29	5	1.9E-05	EU045345

## Conclusion

Development of Leishmania within its sand fly host is largely restricted to the vector midgut. Within the midgut Leishmania begins its development confined within a peritrophic matrix and is subjected to the onslaught of digestive enzymes. Later, they attach to the epithelia to prevent excretion with remnants of the blood meal and detach as they develop into the infective metacyclic form before being transmitted to a suitable host during a subsequent blood meal. The sandfly midgut presents a number of biological barriers the Leishmania parasite must circumnavigate or defeat to proliferate and develop inside the insect vector. Acquiring a better understanding of the molecules present in this organ will illuminate the potential molecular interactions occurring between the Leishmania parasite and the sandfly vector. Comparative transcriptome analysis provides a powerful global approach as demonstrated by the repertoire of molecules identified from a whole organism or from a specific tissue and the generation of new hypotheses from these data. Large scale genome analyses benefit from data generated from transcriptome analyses, for example, by aiding in the annotation of exons and introns.

The results of the present work provide insights into the repertoire of the molecules present in the midgut of the sandfly *P. papatasi*, the natural vector of *L. major*. We identified a variety of molecules and obtained high quality, full-length sequences from many of them. The high quality sequences were deposited at NCBI, significantly augmenting the available midgut-specific coding sequences. A large number of non-annotated sequences were deposited in the EST database for the scientific communities to access these transcripts.

The global changes in sandfly midgut expression profile were assessed by comparing data generated from randomly sequenced midgut cDNA clones obtained from cDNA libraries of adult females fed on sugar only, blood or blood with the addition of *L. major*. Our approach allowed for the identification of transcripts that are induced by blood feeding and likely participate in the digestion of the blood meal and events leading to egg production. Digestion of blood as a nutritional source is complicated by the cellular and molecular response and components of the blood itself, once ingested by the insect vector. Transcripts identified in the *P. papatasi *midgut, such as ferritin, Kazal-type serine protease inhibitors, and GST, are examples of the molecules identified on the gut of this insect. Additionally, the inclusion of a *L. major*-infected midgut cDNA library provides insight into genes potentially regulated by this parasite during its development within the sandfly midgut. The random sequencing approach followed by the *in silico *analysis of the transcript abundance was supported by experimental analyses obtained via real-time PCR.

Overall, this analysis will contribute to the understanding of the molecular interactions between Leishmania and the sandfly vector and may open new avenues for basic research towards the control of this neglected vector-borne disease.

## Methods

### Sandflies

*Phlebotomus papatasi *sandflies (Saudi Arabia strain) were obtained from colonies maintained at Walter Reed Army Institute for Research (WRAIR) and at NIAID-NIH. Three to 5-day old female sandflies were fed either on 20% sucrose solution (sugar fed) or on BALB/c mouse whole blood, via artificial meals [1], with or without the addition of 2 × 10 ^6^*L. major *(V1 strain) amastigotes per ml.

### Messenger RNA extraction and cDNA library construction

*Phlebotomus papatasi *female midguts (10 midguts) were dissected from sugar fed only, from blood fed at 6 h (6 midguts), 24 h, 48 h and 72 h post blood meal PBM (5 midguts each) and from *L. major*-infected at 16 h (3 midguts), 22 h and 96 h (5 midguts each) post infection (p.i.). For blood-fed and for *L. major*-infected, groups of midguts were pooled for RNA extraction. Pooling was done for the sugar-fed group as well. Messenger RNA was purified with the Micro-FastTrack mRNA isolation kit (Invitrogen-Life Technologies, Carlsbad, CA) and 100 ng of mRNA was used to produce a first strand cDNA. A cDNA library, enriched for full-length cDNA, was synthesized using the SMART cDNA library construction kit (Clontech Laboratories, Mountain View, CA). One microgram of double stranded DNA for each original library (sugar-fed, blood-fed, *L. major*-infected) was fractionated using a Chromaspin 1000 column (Clontech Laboratories, Mountain View, CA) into small (S), medium (M) and large (L) transcripts based upon their electrophoresis profile on a 1.1% agarose gel. Pooled fractions were ligated into Lambda TriplEx2 vector (Clontech, Mountain View, CA) and packaged into lambda phage (Stratagene, La Jolla, CA). Individual libraries were plated on LB agar plates in order to achieve roughly 200–300 plaques per 182 mm plates.

### Random sequencing

Unidirectional sequencing of randomly selected clones was completed as previously described [10]. Single, isolated plaques were picked from the plate using sterile wooden sticks and placed into 70 μl of water. Amplification of the cDNA was performed using Platinum PCR SuperMix (Invitrogen), 4 μl template, and primers PT2F1 (AAG TAC TCT AGC AAT TGT GAG C) and PT2R1 (CTC TTC GCT ATT ACG CCA GCT G). PCR amplification products were cleaned using either. Multiscreen PCR cleaning plates (Millipore) or Edge Biosystems PCR cleaning plates and three washes with ultra pure water. The cleaned PCR product was resuspended in 25 μl of water of which 4 μl were used for cycle sequencing with PT2F3 primer (TCT CGG GAA GCG CGC CAT TGT) and either DTCS reaction kit (Beckman) or Big Dye 3.1 (Applied Biosystems). Sequencing reaction products were cleaned using Sephadex G-50 (GE Healthcare) in a multiscreen cleaning plate (Millipore) and analysed using either CEQ8000 (Beckman Coulter) or ABI3700 (Applied Biosystems) DNA sequencing instrument.

### Bioinformatic analysis

Detailed description of the bioinformatic analysis of the data appear in [10,29]. Briefly, prior to analysis the vector sequence was removed from the cDNA nucleotide sequences. Sequence data from the three libraries were grouped together and aligned to generate clusters of contiguous sequences or contigs based on 90% homology over 90 nucleotides, after sequences with more than 5% Ns were discarded. Three frame translations of the consensus sequence of each contig were subjected to comparison using the appropriate BLAST algorithm to the NCBI non-redundant protein database, conserved domain database [30] which contains the eukaryotic clusters of orthologous groups (COG), Simple Modular Architecture Tool (SMART) and Protein Family Database (Pfam), and the Gene Ontology database [31]. Nucleotide sequences were directly compared with two customised databases, mitochondrial and ribosomal RNA (rRNA) nucleotide databases using BlastN. Determination of the presence of a signal secretion peptide or transmembrane helices was accomplished by the submission of sequence peptides to the SignalP server [32] or TMHMM server [33], respectively. The *L. longipalpis *BLAST server was utilized to determine homology between the *P. papatasi *clusters and *L. longipalpis *ESTs [34]. The number of transcripts each library contributed to a particular contig was derived using a custom program, Count Libraries (JMC Ribeiro, personal communication). Comparisons between the sugar-fed and blood-fed midgut cDNA sequences and comparisons between blood-fed and *L. major*-infected midgut cDNA sequences were based on separate Chi-square analysis [35]. The grouped and assembled sequences, BLAST results and signal peptide results were combined in an Excel spreadsheet and the putative function, if any was manually verified and annotated. Sequences were aligned using Clustal X, version 1.83, and converted to graphical aligned sequences using BioEdit, version 7.0.5.3 [36]. Phylogenetic analysis was conducted on amino acid alignments using TREE-PUZZLE, version 5.2, generating trees by maximum likelihood using quartet puzzling to calculate node support [37].

### Quantitative PCR

Quantitative PCR (qPCR) was performed in selected clones using the first-strand cDNA, obtained from 100 ng total RNA isolated from midguts dissected from *P. papatasi *females fed on sugar (unfed) or dissected after a blood meal (24–72 h post blood meal or PBM). cDNAs were synthesized using the 1^st ^Strand cDNA Synthesis kit (Invitrogen, San Diego CA). Transcript levels were measured with SYBR green dye using a LightCycler 2.0 (Roche Diagnostics, Manheim, Germany). For qPCR reactions, samples were subjected to an initial holding step at 95°C for 15 minutes, followed by an amplification step consisting of 35 cycles of 95°C for 10 seconds, 54°C for 20 seconds and 72°C for 20 seconds with a single acquisition. The reaction continued with a single-cycle melting step of 95°C for 10 seconds, 67°C for 30 seconds and 95°C for 10 seconds, prior to cooling for 1 minute. Equal amounts of cDNA were amplified using gene-specific primer sets targeting individual transcripts as well as a *P. papatasi *alpha tubulin, as control or reference transcript. Reactions were routinely done in duplicate. The relative expression ratio of the target transcript and control or reference transcript (fold over control) was calculated using the LightCycler relative quantification software (Roche).

### Semi-quantitative PCR

Semi quantitative RT-PCR reactions were performed with selected transcripts to further demonstrate the differential expression of these genes in *P. papatasi *midgut. In this case, 100 ng of total RNA isolated from midguts dissected from *P. papatasi *females fed on sugar (unfed) or dissected after a blood meal (48 h PBM) were used to synthesize a cDNA using the 1^st ^Strand cDNA Synthesis kit (Invitrogen). PCR reactions were carried out by an initial hot start at 95°C for 5 minutes followed by 25 cycles of 95°C for 30 seconds, 54°C for 1 minute and 72°C for 1.5 minutes and a final extension cycle of 72°C for 5 minutes. PCR products were separated on 1.5% agarose.

## Authors' contributions

JMRO participated in conception of the study, sandfly rearing; construction of cDNA libraries, coordination of the study and in drafting the manuscript. RCJ participated in coordination of the study, bioinformatics analysis, sequence alignment and annotation, phylogenetic analysis and in drafting the manuscript. JMA participated in the bioinformatics analysis and in drafting the manuscript. PGL carried out the rearing of the sand fly colony and drafted part of the manuscript. VMP carried out the sequencing of the transcripts from the three cDNA libraries. SK participated in conception of the study, cDNA library construction, sandfly rearing and drafting the manuscript. JGV participated in conception of the study, participated in its design and coordination, construction of cDNA library and drafting of the manuscript. All authors have read and approved the final manuscript.
